# Oral cleanliness in daily users of powered vs. manual toothbrushes – a cross-sectional study

**DOI:** 10.1186/s12903-019-0790-9

**Published:** 2019-05-29

**Authors:** Waldemar Petker, Ulrike Weik, Jutta Margraf-Stiksrud, Renate Deinzer

**Affiliations:** 1 Department of Medicine, Institute of Medical Psychology, Justus-Liebig-University Giessen, Klinikstr. 29, D-35392, Giessen, Germany; 2 Department of Psychology, Philipps University of Marburg, Gutenbergstr. 18, D-35032 Marburg, Germany

**Keywords:** Preventive dentistry, Oral hygiene, Dental devices, home care, Toothbrushing, Dental plaque, Periodontal diseases, Gingivitis, Behavioral research

## Abstract

**Background:**

Toothbrushing is a daily routine. Still, when adults are asked to manually perform oral hygiene to the best of their abilities, a considerable amount of plaque persists. Little is known about the performance of people who use a powered toothbrush. The present study thus analysed whether the capability to achieve oral cleanliness is better in people for whom powered toothbrushing is a daily routine.

**Methods:**

University students, who either performed powered (*N* = 55) or manual (*N* = 60) toothbrushing for more than 6 months on a daily basis were asked to clean their teeth to the best of their abilities by their own device. Plaque was assessed prior to and immediately after brushing. Furthermore, gingival bleeding, recessions, periodontal pocket depths and dental status were assessed. Oral hygiene performance was video-taped and analyzed with respect to brushing duration, sites of brushing and application of interproximal cleaning devices.

**Results:**

No differences between groups were found with respect to plaque before and after brushing, clinical parameters and overall brushing duration (all *p* > 0.05, all d < 0.156). After brushing, plaque persisted at approximately 40% of the sections adjacent to the gingival margin in both groups.

**Conclusions:**

No advantage of daily powered toothbrushing as compared to daily manual toothbrushing was seen with respect to oral hygiene or clinical parameters. The capability to achieve oral cleanliness was low, irrespective of the type of toothbrush under consideration. Additional effort is thus needed to improve this capability.

**Electronic supplementary material:**

The online version of this article (10.1186/s12903-019-0790-9) contains supplementary material, which is available to authorized users.

## Background

Toothbrushing is an established health behavior aiming to remove dental plaque and thereby to maintain oral health [1]. A representative study shows that in Germany self-reported oral hygiene behavior complies with general recommendations by dental professionals regarding the frequency and duration of daily toothbrushing [2]. Still, the prevalence of gingivitis and periodontitis is considerable in Germany and worldwide [2, 3]. One reason for this observed discrepancy between regularly performed oral hygiene and high prevalence of periodontal disease could be that patients are lacking the appropriate skills to remove plaque. Indeed, a recent series of studies has shown that dental laypeople (i.e. people without any dental background) hardly ever manage to clean more than 30–40% of their gingival margins by means of manual toothbrushing even when they perform to the best of their abilities [4–10]. On the other side, dental professionals achieve oral cleanliness at more than 90% of their gingival margins [11]. This result indicates that effective manual toothbrushing is possible but that dental laypeople appear to have difficulties to acquire this capability. Helping them to acquire it should thus be one of the major aims of preventive activities in dentistry.

Powered toothbrushes represent an alternative to manual toothbrushes and advertisement slogans like “brush like a pro” (e.g. https://www.superdrug.com/brandshop/oral-b; https://www.youtube.com/watch?v=OYldJgTC0H4) suggest that they could increase brushing performance of dental laypeople as compared to manual toothbrushes considerably. This notion is supported by a recent meta-analysis indicating a small though significant advantage of powered vs. manual toothbrushing with respect to oral hygiene and gingivitis [12]. Thus, the question arises whether the capability to achieve oral cleanliness is superior amongst users of powered toothbrushes compared to users of manual toothbrushes? However, no such data is readily available. Although there are numerous studies assessing the effects of powered toothbrushing on plaque after brushing, they do not assess the overall capability to achieve oral cleanliness [13]. Instead, they ask participants to brush as usual or for a certain period of time (1 or 2 min). None of these studies assesses oral cleanliness after best possible performance. To do so was the major aim of the present study. As skills result from training, it is important to examine persons with high proficiency. Thus, daily users should be studied rather than persons who just began powered toothbrushing. On the basis of the trans-theoretical model of health behavior, [14] maintenance of a behavior (and thereby continuity) is assumed after 6 months of continuously showing that behavior. Thus, people should apply the device for at least 6 months. The present study thus assessed the capability to achieve oral cleanliness in daily users of powered toothbrushes who began to continuously perform powered toothbrushing at least 6 months ago. In order to better understand results data assessments were augmented by video analyses of toothbrushing behavior.

A second aim of the present study emerges from results of another study that compared clinical data of daily users of powered vs. manual toothbrushes [15]. No differences were found with respect to clinical data and plaque after brushing. However, in that study people were asked to brush their teeth “as usual”. Thus, nothing is known about differences in oral cleanliness after best possible performance. A second aim of the present study was thus to compare daily users of powered vs. manual tooth brushes in this respect. Furthermore, the study also aimed to prove the replicability of the results of the previous study [15] regarding clinical parameters in daily users of powered vs. manual toothbrushes.

Summarizing, the aims of the present study were 1) to describe oral cleanliness after powered brushing to the best of one’s abilities in daily users of powered toothbrushes, 2) to compare these to cleanliness achieved by manual toothbrushing in daily users of manual toothbrushes and 3) to compare both groups with respect to clinical data and characteristics of toothbrushing behavior.

## Methods

### Participants

Participants were students from the University of Giessen (Hesse, Germany) who were either habitual users of a powered or a manual toothbrush. Inclusion criteria were as follows: 18–30 years of age, at least 20 own teeth. Exclusion criteria were: The use of more than 1/3 of all toothbrushing events per week by another device than the main device (powered vs. manual); study of dentistry/medicine; wear fixed orthodontic appliances or removable dentures; have any physical impairment that affects oral hygiene; undergone dental prophylaxis within the previous four months; pregnancy; antibiotic therapy within the previous six months.

Participants were recruited via postings on the campus, announcements in a local magazine and e-mail circulars of the University’s IT center. Students interested in study participation were contacted by telephone. During this first contact they received detailed information about the study, i.e. toothbrushing while being video-taped and were checked for inclusion and exclusion criteria. Recruitment was stopped when the intended group size (*N* = 50 per group) had been reached in both groups (for the sample size calculation see statistical section). Recruitment and assessment therefore continued for the group whose recruitment target was achieved first and was stopped when the second group also reached the intended size. This was done to avoid bias due to differences in the assessment date or the examiner’s experience and to maintain the blindness of the examiner regarding the condition of the participant (powered vs. manual toothbrush). A diagram showing details of recruitment is provided as additional file (see Additional file 1). Eligible participants were invited.

### General design

This was a quasi-experimental study. The assessments took place in dental examination rooms of the Institute of Medical Psychology, Justus-Liebig-University Giessen, in the period from October 2016 to February 2017. Participants were asked to bring their own toothbrush and to refrain from any oral hygiene behaviour for at least 4 h prior to the scheduled appointment. At arrival in the institute eligibility criteria were assessed a second time by a short interview to ensure that participants fulfilled the exclusion and inclusion criteria. Participants then received detailed information about the study procedure (especially videotaping of tooth-brushing behavior and assessment of clinical data) and the general aim of the study (i.e. to learn more about tooth-brushing behavior and it’s relation to clinical data). They were, however, kept blinded regarding the research questions referring to the comparison of powered vs. manual toothbrush users. After participants gave their informed written consent (for details see Declarations section), demographic data (gender, age) and smoking status were assessed by means of a structured interview and participants were asked when they had begun to use the respective oral hygiene device (manual toothbrush or powered toothbrush) on a regular basis. A dental examiner recorded the dental status and carried out the assessment of plaque and gingivitis (see below). Participants were then led in a separate room and placed in front of a washbasin and a tablet computer with a front camera. The tablet with its camera served as a mirror for the participants and allowed the simultaneous record of the oral hygiene performance. Participants were asked to clean their teeth to the best of their abilities with their own tooth brush and without any time limit. Toothpaste was provided as were several interproximal cleaning devices (dental floss, super floss and interdental brushes in various sizes) in order to allow them to perform proximal hygiene behaviour if they wanted to do so. Participants were left alone while they performed oral hygiene behavior but were video-taped (see below). Plaque and periodontal status were recorded immediately afterwards. Then, photographs of the toothbrushes were taken in order to allow for specification of the type of toothbrush the participants used. Finally, participants were asked to fill in some further psychological questionnaires, which were not an objective of the current analysis and thus would not be further described here.

### Observed oral hygiene behavior

Videos were analyzed by two independent calibrated examiners (WP and PK) by means of the observational software Mangold Interact 16 (Mangold International GmbH, Arnstorf, Germany) with respect to brushing duration (time that toothbrush touches the teeth, without rinsing, spitting, tongue cleaning or breaks) and its distribution across surfaces (occlusal, vestibular, palatinal). For calibration, five videos of individuals not involved in the present study were used. Calibration procedure was considered successful when intra-class correlations (ICCs) were above 0.8. To assess quality of coding after calibration at least 10 videos for each behavioral category were randomly selected and double coded by another person; ICCs of these parameters were all above 0.974. In order to compare groups with respect to their proximal hygiene behavior it was also planned to assess interdental cleaning in detail, however, only 36 participants of the total sample size showed at least some interdental cleaning. Even then, this was confined to only a few proximal spaces. Therefore, no detailed analyses were made.

### Clinical assessment

Clinical assessments were made by a trained dentist (WP), who was blinded to the condition (powered vs. manual toothbrush) of the participants. For plaque and gingivitis assessment, the dental examiner was calibrated by another experienced dentist. For calibration individuals other than those participating in the study were examined. Both dentists examined independently the same individual. Results were then compared. Calibration was considered successful when in each of 5 consecutive individuals 90% of the scorings of the two examiners showed perfect concordance and when the remaining 10% differed by no more than one score. For the assessment of the periodontal status self-calibration was conducted by performing clinical examinations twice with an interval of 5 to 7 days between examinations.

Plaque deposits were stained with a fluorescent disclosing solution (Plaque Test, Ivoclar Vivadent; Germany) and assessed by two indices, the Turesky modification of the Quigley and Hein Index (TQHI; [16] and the Marginal Plaque Index (MPI; [9]. The TQHI assesses plaque at the entire dental crown at two sites per tooth (palatinal and vestibular), and each site is given a score of 0 to 5 (0 = no plaque, 1 = several flecks of stain at the cervical margin of the tooth, 2 = thin continuous band of plaque up to 1 mm at the cervical margin of the tooth, 3 = a band of plaque wider than 1 mm but covering less than 1/3 of the crown, 4 = plaque covering at least 1/3 but less than 2/3 of the crown, 5 = plaque covering at least two thirds of the crown). In previous studies primarily score 1 and 2 were detected, which indicates that the gingival margin is the most relevant area [5, 7, 9]. For this reason, an additional differentiated assessment of the plaque deposits adjacent to the gingival margin was carried out with the help of the MPI [9]. The MPI assesses the presence (score 1) or absence (score 0) of plaque adjacent to the gingival margins within eight sections, four palatinal and four vestibular. Thus, the precise situation of the plaque with respect to the gingival section (i.e., disto-proximal; disto-cervical, mesio-cervical and mesio-proximal) is possible. The MPI has been shown to respond very sensible to changes in oral hygiene behavior [9].

As an indicator of gingivitis the papillary bleeding index [17] modified by Rateitschak [18] was assessed at all palatinal and vestibular surfaces. Each of the surfaces was given a score from 0 to 4 (0 = no bleeding, 1 = single bleeding point, 2 = several bleeding points or thin line, 3 = interdental triangle filled with blood, 4 = profuse bleeding). Probing pocket depths (PPD) and recessions (REC; distance between the gingival margin and the cement-enamel junction) were assessed at six sites per tooth by means of a periodontal probe (UNC-15) marked at each millimeter.

### Statistics

The aims of the present study were 1) to describe oral cleanliness after powered brushing to the best of one’s abilities in daily users of powered toothbrushes, 2) to compare these to cleanliness achieved by manual toothbrushing in daily users of manual toothbrushes and 3) to compare both groups with respect to clinical data and characteristics of toothbrushing behavior. According to the first descriptive statistics for persistent plaque after brushing were computed. For the second and third study aim group comparisons were computed. The primary outcome variable for group comparisons was plaque after oral hygiene to the best of one’s abilities as measured by the MPI. Sample size was determined by the software G-Power [19]. To allow the detection of medium effect sizes with a α-error probability of 5% and a test-power of 80% a sample size of 50 participants per group was needed.

All further statistical analyses were run by IBM SPSS 24.

Group comparisons were run by t-tests and in case of violations of the normal distribution assumption by Mann-Whitney U-Tests. For categorical data Chi^2^-Tests were computed to compare groups. Outlying data were excluded from analyses. A value was considered as being outlying, when it differed by more than 3 standard deviations from the respective group mean. The normal distribution assumption was examined by the Kolmogorov-Smirnov Goodness of Fit test. For clinical parameters t-tests are reported together with Cohen’s d as measure of effect size. GERMAN.

This study conforms to STROBE Guidelines.

## Results

*N* = 118 participants were included in the study. Due to technical problems of their powered tooth-brushes two participants were excluded from the study as they could not complete the assessment. Another participant had to be excluded due to not fulfilling all the inclusion criteria which only became apparent after the evaluation of the questionnaires. Finally, *n* = 60 manual toothbrush (MT) users and *n* = 55 powered toothbrush (PT) users completed the study and provided evaluable data. Of the 55 participants of the PT group, 48 (87%) used a powered toothbrush with oscillating-rotating movements (orPT), six participants (11%) used a sonic toothbrush and one participant used a toothbrush with another movement (2%)., Within those participants using an orPT, *N* = 45 used one of the brand Oral-B and *N* = 3 of other brands. Of the 60 participants of the MT group, 52 (87%) used a toothbrush with vertical bristle (vb) tuft arrangement (*N* = 13 flat-trim, *N* = 39 multilevel) while only eight (13%) used a toothbrush with angled bristle tuft configuration. In order to control for unsystematic variance due to different types of toothbrushes analyses were confined to orPT vs. vbMT users. Additional analyses including all participants are shown in the online appendix (see Additional file 2).

Table 1 shows the group characteristics with respect to age, gender, smoking and duration of the use of the respective device. Except for the duration of the use of the toothbrush groups did not differ significantly regarding any of the variables.

**Table 1:** Group characteristics

	orPT (*n* = 48) Mean ± SD n/n	vbMT (*n* = 52) Mean ± SD n/n	*p*-value
Age	24.69 ± 3.33	24.44 ± 3.08	0.70^a^
Sex (female/male)	30/18	41/11	0.08^b^
Smoking (yes/no)	02/46	03/49	0.99^b^
Duration of use of the respective device (0.5–1 year/1–5 years/> 5 years)	11/23/14	3/11/38	**< 0.001** ^b^

*orPT* habitual users of oscillating-rotating powered toothbrushes, *vbMT* habitual users of manual toothbrushes with vertical bristles, ^*a*^ *t-test* ^b^ *χ*^2^-test

Groups did not differ with respect to periodontal status or gingival recessions, however filled teeth were seen more often in the orPT group as compared to the vbMT group (see Table 2).

**Table 2:** Dental and periodontal status

	orPT (*n* = 48) Mean ± SD n/n	vbMT (*n* = 52) Mean ± SD n/n	*p*-value
*Dental status*			
Number of teeth	28.77 ± 1.88	28.42 ± 1.73	0.34^a^
Filled teeth(0/1–5/> 5 teeth)	14/22/12	23/25/4	**0.05** ^b^
Crowned teeth (0/1–5/> 5 teeth)	43/5/0	51/1/0	0.10^b^
Decayed teeth (0/1–5/> 5 teeth)	48/0/0	48/4/0	0.12^b^
*Periodontal status*			
Recessions^c^ (0/1–5/> 5 sites)	30/12/6	24/21/7	0.23^b^
Periodontal pocket depths > 3 mm (0/1–5/> 5 sites)	28/17/3	33/15/4	0.73^b^

*orPT* habitual users of oscillating-rotating powered toothbrushes, *vbMT* habitual users of manual toothbrushes with vertical bristles, ^a^ t-test, ^b^ *χ*^2^-test, ^c^ measured values were in the range of 1-3 mm, recessions > 3 mm were not detected

Regarding toothbrushing behavior groups did not differ with respect to overall brushing duration though orPT users brushed their palatinal surfaces significantly longer than vbMT users (see Table 3).

**Table 3:** Toothbrushing behavior

	orPT (*n* = 48)^#^ Mean ± SD Median (Min; Max) n/n	vbMT (*n* = 52)^#^ Mean ± SD Median (Min; Max) n/n	effect size d	p
**Overall brushing duration** (s)	225.81 ± 82.18	205.25 ± 70.84	−0.269	0.18*
Occlusal (s)	57.01 ± 28.03	68.58 ± 34.07	0.369	0.07*
Vestibular (s)	100.54 ± 42.40	91.30 ± 37.96	−0.230	0.26*
Palatinal (s)	58.08 (0.24; 123.40)	35.10 (0.00; 129.60)	**–**	**0.01** ^+^
**Application of proximal hygiene devices** (y/n)	20/28	16/36	**–**	0.30^†^

*orPT* habitual users of oscillating-rotating powered toothbrushes, *vbMT* habitual users of manual toothbrushes with vertical bristles, * t-test, ^†^ *χ*^2^-test, ^+^ U-test; ^#^due to exclusion of outlying values in orPT: *n* = 47 for duration of occlusal, vestibular; ^#^due to exclusion of outlying values: *n* = 51 for duration of occlusal surfaces in vbMT

No group differences were found regarding plaque prior to oral hygiene, gingivitis or regarding oral cleanliness achieved after oral hygiene procedure (see Table 4).

**Table 4:** Clinical parameters

	orPT (*n* = 48) Mean ± SD	vbMT (*n* = 52) Mean ± SD	t(98)	effect size d	p
*Plaque before brushing*					
**TQHI** (mean)	1.69 ± 0.63	1.72 ± 0.52	.263	.053	.79
**MPI** (%)	58.75 ± 21.80	60.53 ± 18.57	.440	.088	.66
*Gingivitis*					
**PBI** (mean)^a^	0.16 ± 0.13	0.17 ± 0.10	.323	.065	.75
*Plaque after brushing*					
**TQHI** (mean)	1.19 ± 0.59	1.19 ± 0.43	.057	.011	.95
MPI (%)					
All sections	38.76 ± 20.16	39.69 ± 15.97	.257	.051	.80
Vestibular sections	35.15 ± 23.37	33.62 ± 17.86	−.371	−.074	.71
Palatinal sections	42.36 ± 23.58	45.76 ± 20.13	.778	.156	.44
Cervical sections	35.61 ± 20.73	36.33 ± 15.95	.196	.039	.85
Proximal sections	41.90 ± 20.30	43.04 ± 17.37	.304	.061	.76

*orPT* habitual users of oscillating-rotating powered toothbrushes, *vbMT* habitual users of manual toothbrushes with vertical bristles, ^a^ Due to outliers PBI values refer to *n* = 47 for orPT and *n* = 51 for vbMT

To assess whether the use of proximal hygiene aids masked group differences regarding oral cleanliness after brushing, a further analysis was run with only those participants who did not use any proximal hygiene aids. Still, no difference in plaque after brushing (MPI) was found (t(62) = 0.108; *p* = .914; d = .027).

While the analyses reported so far were confined to orPT vs. vbMT users, similar results were seen when all participants were included and when restrictions regarding outlying values were made (see Additional file 2).

## Discussion

Persons who habitually use a manual toothbrush appear to have difficulties to achieve oral cleanliness even when brushing to the best of their abilities [4–10]. Therefore, the present study aimed to assess whether daily users of a powered toothbrush would show results that are more favorable. According to the data presented here, this is not the case. Although powered toothbrush users brushed their teeth for more than an average of 3 min, the successes of these efforts were limited. Thirty-nine percent of marginal sections showed persistent plaque after participants had brushed their teeth to their best possible performance.

The results of the present study thus support what has been found earlier [4–6, 8, 10]: Young adults appear to have problems to remove all plaque deposits even when they are brushing without any time limit and to the best of their abilities. The present study furthermore extends this result in showing that this problem is not confined to users of a manual toothbrush but exists to the same extent in daily users of a powered toothbrush. Thus, it appears not to be the device as such which might help one to “brush like a pro”. According to a recent study, “to brush like a pro” means to achieve MPI values below 10% after brushing. This is what 75% of a sample of 127 university dentists, dental hygienists and dental students achieved – by manual toothbrushing [11]. The current sample is far away from this degree of oral cleanliness.

However, what hinders effective plaque removal in dental laypeople? Video observation provides important insights regarding brushing behavior. In the present study, participants brushed their teeth for a remarkably long duration (more than 3 min). Furthermore, PT users spent approximately one minute brushing palatinal surfaces, almost twice as long as MT users. Still there was no advantage regarding oral cleanliness. The correct execution of brushing movements does not seem to be the issue, considering that the toothbrush itself moves the bristles. The problem could thus be the exact brushing localization with respect to the gingival margin. Indeed, a representative survey on periodontitis-related knowledge shows that 50% of young adults believe that brushing of occlusal surfaces is the most important when it comes to the prevention of periodontitis [20]. Due to limitations of sight in video analyses it was not possible to assess whether the bristles of the brushes reached tooth sections adjacent of the gingival margin or not. Still, plaque data indicated that participants neglected the gingival margins. This appears to be the case regardless of the device used. In comparison to plaque levels of daily users of manual toothbrushes, no advantage of powered toothbrushing could be observed. The percentage of marginal sections showing persistent plaque immediately after manual toothbrushing was 41%. This represents only a small and non-significant difference of 2% to powered toothbrushing. Furthermore, plaque persistence was more pronounced at the palatinal and proximal surfaces. But again groups did not differ regarding these locations. This is remarkable as users of powered toothbrushes brushed their palatinal surfaces significantly longer than manual toothbrush users. TQHI data further indicate that these results are not confined to the gingival margin. Groups also did not differ with respect to the TQHI, which assesses plaque deposits throughout the crown. With these results in mind it is not surprising that groups do not differ in oral hygiene before brushing, in gingivitis, periodontal status or dental status.

At the first glance, this lack of a difference between daily users of powered vs. daily users of manual toothbrushes appears to be surprising. According to a recent Cochrane Review powered toothbrushes appear to reduce plaque and gingivitis more effectively than manual toothbrushes in the short and long term [12]. In order to better understand the seemingly contradictory results, it is worth taking a closer look at the studies and outcome variables included in the meta-analysis [12]. First of all, plaque levels assessed immediately after brushing were not taken into account. Thus, no information is provided regarding the capability of the participants to achieve oral cleanliness. Also, study arms differed systematically with respect to the toothbrushing instructions being provided. Participants assigned to a powered toothbrush either received written instructions according to the manufacturer or were shown the correct use of the brush by study staff [21–40]. On the other hand, participants assigned to a manual toothbrush either were asked to brush their teeth as they normally do [21–24], or were instructed to apply the (modified) Bass technique [25–40]. However, recent RCTs question the efficacy of the modified Bass technique [5]. Finally, by enrolling in the study, participants were also provided with a new toothbrush – either a cheap (i.e. manual) or an expensive (i.e. powered) one. This difference in prices could have changed hygiene behavior selectively in favor of the more expensive one for two reasons: First, because participants in the powered group appreciated being allocated to the more expensive device, secondly, because of demand characteristics. Demand characteristics are a well-studied phenomenon responsible for experimental artifacts. Participants of research tend (even unconsciously) to behave into the direction of the supposed hypothesis of the researcher [41]. In the present case a supposition in favor of the more expensive device suggests itself. Even though these systematic group differences are partly inevitable when planning a RCT comparing powered vs. manual toothbrushing they might cause bias in favor of the powered toothbrushes. These might account for the statistically significant though small differences between PT and MT application which have been described in the above mentioned meta-analysis [12].

It is thus important to run studies which avoid these biases. The present study aimed to do so by including only participants who had decided to brush their teeth by a manual or powered toothbrush deliberately instead of being randomly allocated to one of the two devices. This should have reduced the potential biases named above. Though the missing randomization might have resulted in other systematic group differences, the examined groups were homogenous and comparable with respect to a number of potential confounding variables, like gender, age, and smoking status. While there were some group differences regarding brushing behavior, these pointed into the direction of an advantage of the powered brushes which still was not detectable. Thus, the results of the present study question whether the advantage found for powered toothbrushes in experimental settings would persist in common users outside of experiments. To the best knowledge of the authors of the present study, there is only one other study which compared daily users of an oscillating-rotating powered toothbrush (who did not start powered toothbrushing in the context of an experiment) to daily users of a manual toothbrush [15]. In that study, participants were asked to clean their teeth as they normally do and were not allowed to use any proximal hygiene devices. As in the present study, groups did not differ with respect to plaque levels after brushing. While the authors of that study argued that plaque removal in the powered toothbrush group was significantly better, this advantage resulted from a (non-significant) disadvantage regarding plaque levels prior to toothbrushing which was worse in powered vs. manual toothbrush users. Furthermore, as in the present study, groups of that study did not differ regarding gingival bleeding, gingival recessions and probing pocket depths. Similar results were obtained by Al-Maliky et al. [42] who assessed a group of healthy Dutch students and related their self-reported oral hygiene behavior to gingivitis. Again, no significant difference with respect to gingivitis between users of powered and manual toothbrushes was found. These results question the advantage of powered toothbrushes in daily use and call for further studies to investigate this issue.

Summarizing, the present study found that daily users of powered toothbrushes were not capable to achieve oral cleanliness even if they were asked to perform oral hygiene to the best of their abilities. Instead, they showed the same deficits like a comparable group of daily users of a manual toothbrush. Furthermore, no differences between daily users of powered vs. manual toothbrushes were found with regards to other clinical parameters. This result is confirmed by other studies on daily users of powered toothbrushes who started powered toothbrushing outside an experimental setting. These results indicate that deficits regarding manual oral hygiene capabilities cannot be easily overcome by merely changing to a powered device. Instead, further effort is needed. A first step might be to underline the necessity of brushing systematics in order to not neglect any surface and the significance of the regions adjacent to the gingival margin when it comes to the prevention of periodontal disease.

The present study has its strengths not only in the thorough clinical investigation of a large sample but also in a detailed analysis of oral hygiene behavior and the control of several potential confounders (blinding of clinical examiner, no disclosure of information regarding hypothesis to participants). Another advantage is that it analysed the capabilities of daily users of powered vs. manual devices. It thus might reflect clinical reality better than studies with new users of these devices or with users who began powered toothbrushing in the context of an experiment. However, some limitations must also be taken into consideration. The potential limitation resulting from the quasi-experimental study design has already been discussed. Another limitation is that results might be confined to specific types of toothbrushes. Even though there was no restriction in recruitment in this respect, the majority of powered toothbrush users used an oscillating-rotating toothbrush (and within this group one brand predominated) and manual toothbrushes mostly showed a vertical bristle tuft arrangement. Thus, it is unclear how the current results apply to other types of toothbrushes. Furthermore, though the sample reflects daily users it is confined to daily users who are university students, a young, highly educated, and periodontally healthy group. Future studies should assess the generalizability to other groups. Finally, one might criticize, that brushing to the best of one’s ability instead of brushing as ‘normal’ was investigated in this study. This, however, was exactly the aim of the study, as it is important to figure out what patients can achieve by applying maximum effort (for a comparison of “normal” brushing behavior to brushing to the best of one’s ability see [10]). Only on this basis, one can conclude whether hygiene deficits result from a lack of motivation or rather from a lack of skills. This is of crucial importance in the choice of treatment. The present investigation indicates that oral hygiene deficits in young adults reflect at least in part deficits in skills.

## Conclusion

Concluding, the present study confirms earlier results that young adults have difficulties to achieve oral cleanliness even if they brush their teeth to the best of their abilities. While findings from former studies were confined to manual toothbrushing, the current research reveals that they also apply to powered toothbrushing. Thus, attempts to improve oral hygiene skills should rely on other factors than merely changing the device. Behavioral analyses indicate that all measures that improve the completeness of brushing so that all teeth and all surfaces are brushed might be useful. Furthermore, dental laypeople might not yet sufficiently know the significance of plaque removal at the gingival margin. This is what clinical data and by data from previous surveys suggest [4–9]. However, more research is needed to understand the origin of oral hygiene deficits in dental laypeople. One approach could be to compare their behavior to that of dental professionals since those achieve very good oral cleanliness [11]. This could give important insights about how oral hygiene deficits in dental laypeople could be addressed best.

## Additional files

Additional file 1: Flow diagram. (DOCX 34 kb)

Additional file 2: Clinical parameters when all participants are included into analyses. (DOCX 19 kb)

## Data Availability

The datasets used and/or analysed during the current study are available from the corresponding author on reasonable request. For privacy reasons, however, individual data allowing for the identification of participants (e.g. videos) cannot be made available.

## Abbreviations

ICC: Intraclass correlation
MPI: Marginal Plaque Index
MT: Manual toothbrush
or: Oscillation-rotation
PBI: Papillary bleeding index
PK: Pascal Kellner (assistant during analyses)
PPD: Probing pocket depths
PT: Powered toothbrush
RCT: Randomized controlled trial
SD: Standard deviation
TQHI: Turesky modification of the Quigley and Hein Index
vb: Vertical bristles
WP: Waldemar Petker (author)
