# Supplementary material for: Oral cleanliness in daily users of powered vs. manual toothbrushes – a cross-sectional study
Source: BMC Oral Health. 2019 May 29;19:96. doi: 10.1186/s12903-019-0790-9 (PMC6542008; doi:10.1186/s12903-019-0790-9)
Supplement: Supplementary file 1 — Flow diagram. (DOCX 34 kb) [file 12903_2019_790_MOESM1_ESM.docx]

**Assessed for eligibility**: n=235

**Excluded**: n=118
- not meeting inclusion criteria (n=64)*
- no time, no further interest (n=30)
- required sample size reached (n=24)

**Included**: n =117

**Manual toothbrush**: n=62

examined: n=60
excluded (incomplete data due to technical problems): n=2

**Powered toothbrush**: n=55

Examined: n=55

* reasons for exclusion:

- older than 30 years: n=3;
- Not university student/ studying medicine: n=4;
- fixed orthodontic appliances: n=3
- dental prophylaxis within the previous four months: n=18
- antibiotic therapy within the previous six months: n=24
- using the respective toothbrush type for less than 6 months: n=12
