# Supplementary material for: Oral cleanliness in daily users of powered vs. manual toothbrushes – a cross-sectional study
Source: BMC Oral Health. 2019 May 29;19:96. doi: 10.1186/s12903-019-0790-9 (PMC6542008; doi:10.1186/s12903-019-0790-9)
Supplement: Supplementary file 2 — Clinical parameters when all participants are included into analyses. (DOCX 19 kb) [file 12903_2019_790_MOESM2_ESM.docx]

| **Clinical parameters when all participants are included into analyses** | | | | | | | | |
| --- | --- | --- | --- | --- | --- | --- | --- | --- |
|  | | **PT (n=55)**  Mean ± SD | **MT (n=60)**  Mean ± SD | **t(113)** | **d** | **95% lower CL** | **95% upper CL** | **p** |
| *Plaque before brushing* | | | | | | | | |
| **TQHI** (mean) | | 1.69 ± 0.61 | 1.77 ± 0.51 | .747 | .139 | -.227 | .506 | .46 |
| **MPI** (%) | | 58.35 ± 21.18 | 62.29 ± 18.68 | 1.060 | .198 | -.169 | .565 | .29 |
| *Gingivitis* | | | | | | | | |
| **PBI** (mean) | | 0.19 ± 0.22 | 0.18 ± 0.12 | -.213 | -.040 | -.406 | .326 | .83 |
| *Plaque after brushing* | | | | | | | | |
| **TQHI** (mean) | | 1.21 ± 0.58 | 1.24 ± 0.44 | .300 | .056 | -.310 | .422 | .77 |
| **MPI** (%) | |  |  |  |  |  |  |  |
|  | All sections | 39.26 ± 19.66 | 41.42 ± 16.74 | .637 | .119 | -.247 | .485 | .53 |
|  | Vestibular sections | 35.53 ± 23.30 | 35.19 ± 17.87 | -.089 | -.017 | -.383 | .349 | .93 |
|  | Palatinal sections | 42.99 ± 22.63 | 47.66 ± 21.41 | 1.136 | .212 | -.155 | .579 | .26 |
|  | Cervical sections | 36.07 ± 20.35 | 38.18 ± 16.82 | .608 | .113 | -.253 | .480 | .55 |
|  | Proximal sections | 42.45 ± 19.67 | 44.67 ± 17.84 | .634 | .118 | -.248 | .485 | .53 |
| PT: powered toothbrush; MT: manual toothbrush | | | | | | | | |
